# Conditional Fgfr1 Deletion in GnRH Neurons Leads to Minor Disruptions in the Reproductive Axis of Male and Female Mice

**DOI:** 10.3389/fendo.2020.588459

**Published:** 2021-02-19

**Authors:** Cynthia Dela Cruz, Cassandra A. Horton, Kelsey N. Sanders, Nathan D. Andersen, Pei-San Tsai

**Affiliations:** Department of Integrative Physiology, University of Colorado, Boulder, CO, United States

**Keywords:** gonadotropin-releasing hormone neurons, fibroblast growth factor receptor 1, conditional deletion, hypothalamic-pituitary gonadal axis, congenital hypogonadotropic hypogonadism, Kallmann syndrome

## Abstract

In humans and mice, inactivating mutations in fibroblast growth factor receptor 1 (*Fgfr1*) lead to gonadotropin-releasing hormone (GnRH) deficiency and a host of downstream reproductive disorders. It was unclear if Fgfr1 signaling directly upon GnRH neurons critically drove the establishment of a functional GnRH system. To answer this question, we generated a mouse model with a conditional deletion of *Fgfr1* in GnRH neurons using the Cre/loxP approach. These mice, called *Fgfr1cKO* mice, were examined along with control mice for their pubertal onset and a host of reproductive axis functions. Our results showed that *Fgfr1cKO* mice harbored no detectable defects in the GnRH system and pubertal onset, suffered only subtle changes in the pituitary function, but exhibited significantly disrupted testicular and ovarian morphology at 25 days of age, indicating impaired gametogenesis at a young age. However, these disruptions were transient and became undetectable in older mice. Our results suggest that Fgfr1 signaling directly on GnRH neurons supports, to some extent, the reproductive axis function in the period leading to the early phase of puberty, but is not critically required for pubertal onset or reproductive maintenance in sexually mature animals.

## Introduction

The hypothalamic-pituitary-gonadal (HPG) axis is essential for the initiation and maintenance of fertility. The most upstream hormone of this axis is gonadotropin-releasing hormone (GnRH). GnRH neurons originate in the olfactory placode (OP) at the tip of the nose and must migrate from the OP to the forebrain destinations, where they target their axons towards the median eminence to release GnRH (1, 2). By acting on the pituitary, GnRH stimulates the secretion of gonadotropins, luteinizing hormone (LH), and follicle-stimulating hormone (FSH), which subsequently act on the gonads to stimulate gametogenesis and steroidogenesis (3).

Congenital hypogonadotropic hypogonadism (CHH) is a rare genetic disorder caused primarily by a deficiency in the GnRH system (4). CHH afflicts 1 in 10,000–100,000 individuals and is more prevalent in men (5). CHH is clinically characterized by absent or partial puberty, low circulating gonadal steroids and infertility (4, 6, 7). One particular form of CHH is associated with anosmia or hyposmia (absent or reduced sense of smell). This form of CHH, called Kallmann syndrome (KS), is causally linked to inactivating mutations on genes critical for the development of the olfactory region (8). These mutations likely lead to the simultaneous disruption of the olfactory system development and genesis and/or migration of GnRH neurons within the nose, culminating in the dual failure of the olfactory and reproductive systems in afflicted individuals. Whereas other forms of CHH can have different etiologies, reproductive deficits in KS are invariably caused by the developmental disruption of the GnRH system (8).

An important gene causally linked to KS is *fibroblast growth factor receptor 1* (*Fgfr1*) (9, 10). Supporting a critical role of *Fgfr1* in GnRH system development, homozygous *Fgfr1* hypomorphic mice suffered a 90% reduction in GnRH neurons at birth (11). *Fgfr1* is widely expressed in the olfactory region housing the GnRH progenitor cells (12, 13), within the migratory path of GnRH neurons (14), and in GnRH neurons themselves (13). It is generally agreed that Fgfr1 signaling is necessary for the formation of the surrounding olfactory structures needed to support GnRH neurons, but whether Fgfr1 signaling directly on GnRH neurons played a critical role in their development and function remained unclear and posed an interesting question in the mechanism of disease leading to KS.

In the present study, we generated a mouse with a conditional deletion of *Fgfr1* in GnRH neurons (*GnRH-Cre^+/-^: Fgfr1^flox/flox^*) using the Cre/LoxP technology. Using these mice, we aimed to understand the impact of *Fgfr1* deficiency directly upon GnRH neurons and examine if such deficiency could irreparably damage the GnRH system to impair the HPG axis of postnatal male and female mice. Our results showed that the conditional deletion of *Fgfr1* in GnRH neurons impacted several early pubertal HPG parameters in males and females but had no effect on postpubertal animals. Further, the conditional deletion of *Fgfr1* did not lead to GnRH deficiency seen in the global *Fgfr1*-deficient mice (11). Our results suggest that Fgfr1 signaling directly on GnRH neurons supports, to some extent, the early pubertal HPG axis but is not required for the HPG function in sexually mature animals.

## Materials and Methods

### Transgenic Animals

The Cre/LoxP technology was used to conditionally delete the functional *Fgfr1* allele only in GnRH neurons. For this, a heterozygous male mouse with a GnRH promoter driving the expression of *Cre* (*GnRH-Cre^+/-^*) (15) was bred with a homozygous female mouse with loxP sites flanking Exon 4 of the *Fgfr1* gene (*Fgfr1^flox/flox^*) (16). This mating generated F1 offspring of various genotypes, including *GnRH-Cre^+/-^:Fgfr1^flox/-^*. The F1 male *GnRH-Cre^+/-^:Fgfr1^flox/-^* were backcrossed with female *Fgfr1^flox/flox^* to generate the experimental genotype with a conditional deletion of *Fgfr1* in GnRH neurons (*GnRH-Cre^+/-^:Fgfr1^flox/flox^*). These animals were abbreviated as *Fgfr1cKO* mice. Controls were *GnRH-Cre^-/-^: Fgfr1^flox/flox^* littermates of *Fgfr1cKO* and were abbreviated as control mice. The day of birth was designated as postnatal day (PN) 0. All mice were housed in an animal facility at the University of Colorado Boulder campus under a 12-h light:12-h dark cycle and fed *ad libitum*. At PN20, pups were weaned and genotyped by polymerase chain reaction (PCR) of genomic DNA isolated from tail biopsies for the presence of *GnRH-Cre* and *Fgfr1^flox^* sequences. Primers sequences were: *5’CGGACAGAAGCATTTTCCAG* (forward) and *5’ACAGGTGTCTGTCCCATGTCT* (reverse) for *GnRH-Cre*, and *5’GGACTGGGATAGCAAGTCTCTA* (forward) and *5’GTGGATCTCTGTGAGCCTGAG* (reverse) for *Fgfr1^flox^*. Both males and females were used in this study. All males were sacrificed on PN25 and PN60. All females were sacrificed on PN25 and on diestrus around PN60 (± 5 days). All animal procedures were approved by the Institutional Animal Care and Use Committee.

### Validation of Animal Models

Parental *GnRH-Cre^+/-^* and *Fgfr1^flox/flox^* lines were validated independently to test the ability of their *Fgfr1cKO* offspring to harbor tissue-specific deletion of *Fgfr1*. The *GnRH-Cre^+/-^* line was validated by mating a *GnRH-Cre^+/-^* male with a female universal Cre-responder (Ai9) harboring a loxP-flanked STOP cassette upstream of *tdTomato* (17). The resulting offspring positive for both *GnRH-Cre^+/-^* and *tdTomato* were harvested for their brains and processed for GnRH immunohistochemistry as previously described (13). Alexa 488 (Invitrogen) was used to mark GnRH-positive neurons. The presence of tdTomato- and Alexa488-positive neurons was examined and photographed using an Olympus epifluorescence microscope equipped with the corresponding filter cube.

The *Fgfr1^flox/flox^* line was validated by mating a *Fgfr1^flox/flox^* male with a universal deleter female (Sox2-Cre) expressing Cre recombinase under the control of the mouse Sox2 promoter (18) or a wildtype female. Genomic DNA was purified from the resulting offspring and subject to quantitative PCR (qPCR) for the relative quantification of genomic *Fgfr1* Exon 4 according to the protocol described below under the “*RNA isolation, cDNA synthesis and qPCR*” section. The qPCR primers used were *5’GTTCAAGTGCCCGTCGAGTC* (forward) and *5’ACGCGTACCTTGTAGCCTCC* (reverse) for *Fgfr1* Exon 4, and *5’CACGTGGGCTCCAGCATT* (forward) and *5’TCACCAGTCATTTCTGCCTTTG* (reverse) for *Apob*, a single-copy housekeeping nuclear gene (19).

### Pubertal Assessments

Mice were checked daily starting at PN21 for balanopreputial separation (BPS) in males and vaginal opening (VO) in females to gauge the age of pubertal onset. One day after VO, vaginal smears were performed daily to determine the day of first estrus.

### Tissue Harvest

On PN25 and PN60 (± 5 days for females), animals were lightly anesthetized with isoflurane vapor, weighed, and sacrificed by decapitation. Trunk blood was collected, left to coagulate for 1–2 h, and cleared by centrifugation to generate serum. All serum samples were stored frozen at -20°C until LH radioimmunoassay (RIA).

Brains were dissected and blocked to generate the preoptic area (POA) fragment by an anterior cut at the caudal border of the olfactory bulbs, a posterior cut at 1mm caudal to the optic chiasm, and a dorsal cut to remove the cortex. The remaining brain was blocked to generate the hypothalamic fragment by a posterior cut at the anterior border of the mammillary body, two sagittal cuts along the lateral borders of the hypothalamus, and a dorsal cut to remove the cortex (20). The POA, hypothalamic fragment and pituitary were frozen at -70°C until the measurement of *GnRH* mRNA in the POA by qPCR, and of GnRH and gonadotropin protein levels in the hypothalamic fragment and pituitary by RIAs. Gonads were dissected, weighed, and immersion-fixed in Bouin’s fixative for 24 h at room temperature then stored in 70% ethanol until histological analysis. Seminal vesicle (SV) mass, uterine mass, and anogenital distance were also recorded at the time of sacrifice.

### GnRH, LH, and FSH Radioimmunoassays (RIAs)

Hypothalamic GnRH content was measured by an RIA using a GnRH1-specific antiserum (R1245, provided by T. M. Nett at Colorado State University). Detailed protocol for the GnRH RIA was described elsewhere (21). The detection limit was 1.87 pg/tube. The intra- and inter-assay coefficients of variation were 7.3 and 5.0%, respectively. Serum LH and pituitary contents of LH and FSH were measured using rat LH and FSH RIAs previously described (21). The detection limit for the LH RIA was 0.06 ng/ml, and the intra- and inter-assay coefficients of variation were 9.3 and 10.1%, respectively. For the FSH RIA, the detection limit was 1 ng/ml, and intra- and inter-assay coefficients of variation were 6.6 ± 2.4% and 8.9± 1.5%, respectively. Only LH RIA was performed on serum samples because low sample volume precluded the measurement of both gonadotropins.

### RNA Isolation, cDNA Synthesis, and qPCR

Total RNA from frozen POA fragments was isolated using Trizol reagent (Thermo Fisher Scientific), and first-strand cDNA was synthesized using the QuantTect Reverse Transcription kit (Qiagen). Relative quantification of mouse *GnRH* mRNA was performed by qPCR using *hypoxanthine guanine phosphoribosyl transferase* (*HPRT*) as a housekeeping gene. Primer sequences were: *5’TCAGGGATCTGCGAGGAC* (forward) and *5’GGGCCAGTGCATCTACATC* (reverse) for *GnRH*, and *5’AGCAGTACAGCCCCAAAATGG* (forward) and *5’TGCGCTCATCTTAGGCTTTGT* (reverse) for *HPRT*. All amplifications were performed for 40 cycles with an annealing temperature of 60°C. The relative expression values were calculated using the 2^-ΔΔCT^ method (22).

### Gonadal Histology and Analysis

Fixed gonads were dehydrated in increasing concentrations of ethanol, cleared in Histoclear (National Diagnostics), and embedded in paraffin. Serial sections were cut coronally at 12-μm thickness using a rotary microtome, mounted on gelatin-subbed slides and stained with hematoxylin and eosin before dehydration and coverslipping. For testicular analysis, every fifth section per testis was scored for a total of 10 sections per animal. Each section was divided into the following five fields: top, bottom, right, left, and middle. Within each field, four randomly selected seminiferous tubules (ST) were analyzed for mean (1) ST area, (2) ST perimeter, (3) percent open ST, and (4) percent ST with mature spermatozoa. Morphometric analyses were performed using the Olympus cellSens software.

Ovaries were analyzed for the numbers of primordial follicles, preantral follicles, antral follicles, and corpora lutea. A primordial follicle was defined as an oocyte surrounded by one layer of granulosa cells with no visible space between granulosa cells and the oocyte. A preantral follicle was identified as an oocyte with two or more layers of granulosa cells with narrow or no visible space between granulosa cells and the oocyte. An antral follicle was recognized by the presence of a fragmented or continuous antral cavity within the granulosa cell layers (23). The analysis was performed by an investigator blind to the identity of the slides. The quantification of ovarian structures was conducted on every fifth section, and sections from the entire ovary were scored.

### Fertility Assessment

To evaluate female fertility in an age range encompassing PN60, each control or *Fgfr1cKO* female was paired on PN40 with a control male with a proven history of fertility. Time to first litter, total number of pups born, days between litters, and pups per litter were recorded. Fertility was monitored between PN40 to PN240 to allow sufficient time for the production of multiple litters.

### Statistical Analysis

Gonadosomatic index (GSI) for males and females as well as SV somatic index (SVSI) and uterine somatic index (USI) were calculated by dividing the mass of the respective organ by the body mass. All statistical analyses were performed using Prism (GraphPad). Student’s *t*-test was performed for two-group comparisons. All remaining analyses utilized two-way ANOVA followed by Tukey’s post-hoc test. The level of significance was defined as *P* < 0.05. All data were presented as mean ± SEM.

## Results

To support the tissue-specific deletion of *Fgfr1* in *GnRH-Cre*^+/-^: *Fgfr1^flox/flox^* mice, we independently validated the parental *GnRH-Cre*^+/-^ and *Fgfr1^flox/flox^* lines. The expression of *GnRH-Cre*^+/-^ resulted in tissue-specific recombination as shown by the simultaneous presence of tdTomato and GnRH immunoreactivity (**Supplementary Figure 1A**). In addition, a universal deleter mouse (Sox2-Cre) was able to excise ~50% of *Fgfr1* Exon 4 in offspring harboring a *Fgfr1^flox^* allele (**Supplementary Figure 1B**).

To characterize the *Fgfr1cKO* mice, we first assessed several parameters of pubertal onset, including the age of BPS in males and the age of VO and first estrus in females. *Fgfr1cKO* males did not show altered timing of BPS compared to controls (**Supplementary Table 1**). For females, neither the age of VO nor first estrus was altered in *Fgfr1cKO* compared to control females (**Supplementary Table 1**).

Gross anatomical parameters were assessed in PN25 and PN60 control and *Fgfr1cKO* mice. There were no significant differences between genotypes in body mass, anogenital distance, GSI, SVSI, and USI in males (**Supplementary Figure 2**) and females (**Supplementary Figure 3**). All parameters, except GSI in females (**Supplementary Figure 3B**), exhibited age-dependent increases (**Supplementary Figures 2, 3**). No genotype x age interaction was observed in any parameter.

**Figure 1:**
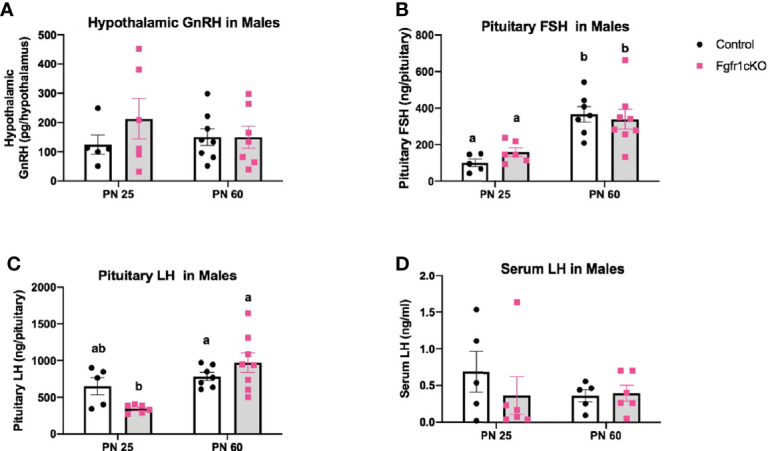
Hypothalamic GnRH **(A)**, pituitary FSH **(B)**, pituitary LH **(C)**, and serum LH **(D)** in PN25 and PN60 control and *Fgfr1cKO* male mice. Each bar represents mean ± SEM, *n* = 5–8. Two-way ANOVA results are indicated in Results section. Different letters above the bars indicate P < 0.05 by post-hoc test.

**Figure 2:**
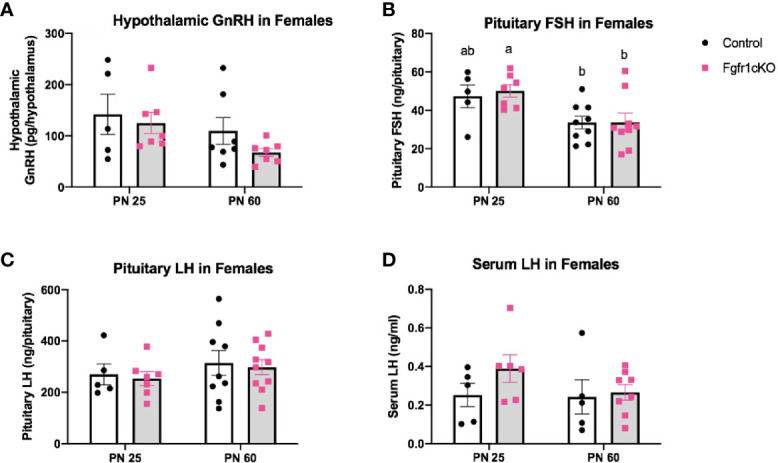
Hypothalamic GnRH **(A)**, pituitary FSH **(B)**, pituitary LH **(C)**, and serum LH **(D)** in PN25 and PN60 (± 5 days) control and *Fgfr1cKO* female mice. All PN60 females were sacrificed on diestrus. Each bar represents mean ± SEM, *n* = 5–10. Two-way ANOVA results are indicated in Results section. Different letters above the bars indicate P < 0.05 by post-hoc test.

**Figure 3:**
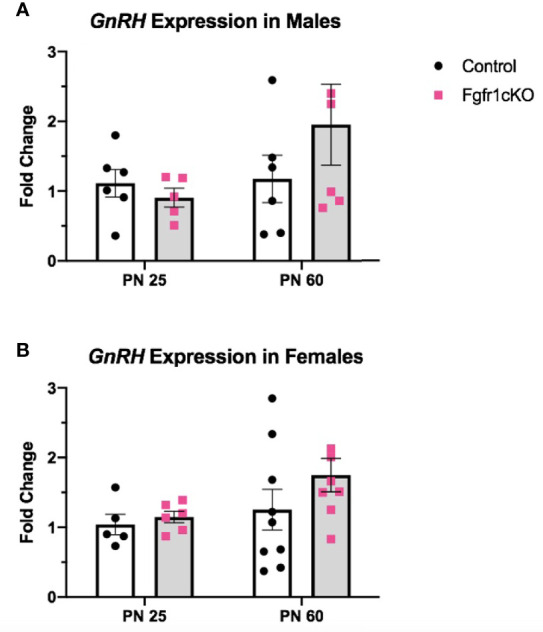
Relative expression of *GnRH* in PN25 and PN60 control and *Fgfr1cKO* males **(A)** and females **(B)**. Each bar represents mean ± SEM, *n* = 5–9. No significant differences were observed between genotypes and age groups.

Next, we examined, by RIA, hypothalamic GnRH, pituitary FSH and LH, and serum LH in both genotypes at different ages and in both sexes. For males, two-way ANOVA showed no significant effect of age, genotype or genotype x age interaction on hypothalamic GnRH (**Figure 1A**). Pituitary gonadotropins exhibited a significant age effect ([*F*(1,22)=25, *P*<0.0001] for FSH; [*F*(1,22)=14, *P*=0.0011] for LH), but genotype x age interaction only on pituitary LH ([*F*(1,22)=6, *P*=0.0225] (**Figures 1B, C**), and no genotype effect on either pituitary LH or FSH (**Figures 1B, C**). Post-hoc test showed significantly elevated pituitary FSH in older males compared to younger mice (**Figure 1B**) and significantly reduced pituitary LH in PN25 *Fgfr1cKO* males compared to older mice of both genotypes (**Figure 1C**). No significant effects of age, genotype, or age x genotype interaction were observed for serum LH (**Figure 1D**). For females, two-way ANOVA showed no significant effect of genotype, age, or genotype x age interaction on hypothalamic GnRH (**Figure 2A**), pituitary LH (**Figure 2C**), and serum LH (**Figure 2D**). Pituitary FSH exhibited a significant age effect [*F*(1,26)=11.7, *P*=0.0021] but no genotype effect or genotype x age interaction (**Figure 2B**). Post-hoc test showed significantly elevated pituitary FSH in PN25 *Fgfr1cKO* females compared to older mice of both genotypes (**Figure 2B**). Lastly, we examined *GnRH* transcript levels by qPCR and found no significant effect of age, genotype, or genotype x age interaction in males and females (**Figure 3**).

**Figure 4:**
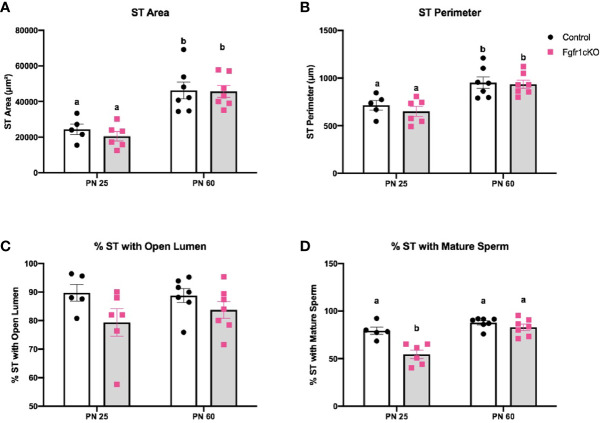
Testicular morphology in PN25 and PN60 control and *Fgfr1cKO* male mice. ST area **(A)**, ST perimeter **(B)**, %ST with open lumen **(C)**, and % ST with mature spermatozoa **(D)** were scored by an investigator blind to the identity of the samples. Each bar represents mean ± SEM, *n* = 5-7. Two-way ANOVA results are indicated in Results section. Different letters above the bars indicate P < 0.05 by post-hoc test.

**Figure 5:**
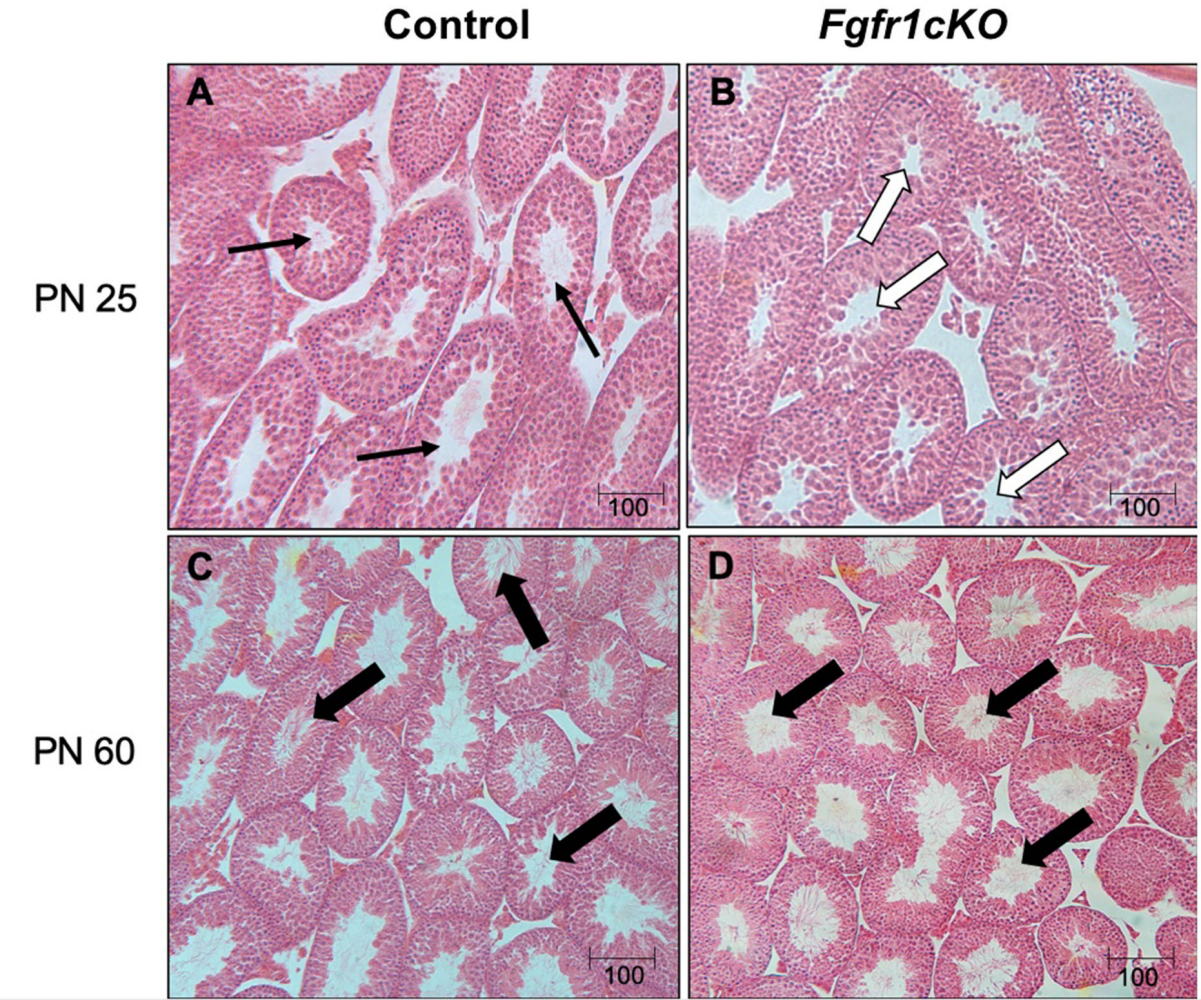
Representative photomicrographs of testicular morphology in PN25 and PN60 control and *Fgfr1cKO* males. For PN25 **(A, B)**, thin arrows in the control testis **(A)** point to open ST with visible spermatozoa, and open arrows in the *Fgfr1cKO* testis **(B)** point to open ST with no visible spermatozoa. For PN60 **(C, D)**, thick arrows point to ST with abundant mature spermatozoa in control **(C)** and *Fgfr1cKO* males **(D)**. Scale bars = 100 µm.

For the assessment of male gonadal histology, two-way ANOVA revealed a significant effect of age on ST area [*F*(1,21)=40.38, *P*<0.0001] (**Figure 3A**) and ST perimeter [*F*(1,21)=24.65, *P*<0.0001] (**Figure 4B**), but not genotype effect or genotype x age interaction on these two parameters (**Figures 4A, B**). Post-hoc test showed significant increases in ST area and perimeter in PN60 compared to younger animals (**Figures 4A, B**). More complex effects were observed for %ST with open lumen and mature spermatozoa (**Figures 4C, D**). Two-way ANOVA revealed a significant genotype effect [*F*(1,21)=5.16, *P*=0.03] on %ST with open lumen (**Figure 4C**), but individual differences were not detected by post-hoc test (**Figure 4C**). Importantly, two-way ANOVA revealed a significant effect of genotype [*F*(1,21)=18.17, *P*=0.0003], age [*F*(1,21)=27.94, *P*<0.0001], and genotype x age interaction [*F*(1,21)=8.2, *P*=0.0093] on %ST with mature spermatozoa (**Figure 4D**). Post-hoc test showed a significant reduction in %ST with mature spermatozoa in PN25 *Fgfr1cKO* males compared to PN25 controls and older males (**Figure 4D**). Photomicrographs (**Figure 5**) demonstrated the presence of ST lacking mature spermatozoa in PN 25 *Fgfr1cKO* males (**Figure 5B**) compared to spermatozoa-filled ST in PN25 controls and older males (**Figures 5A, C, D**).

**Figure 6:**
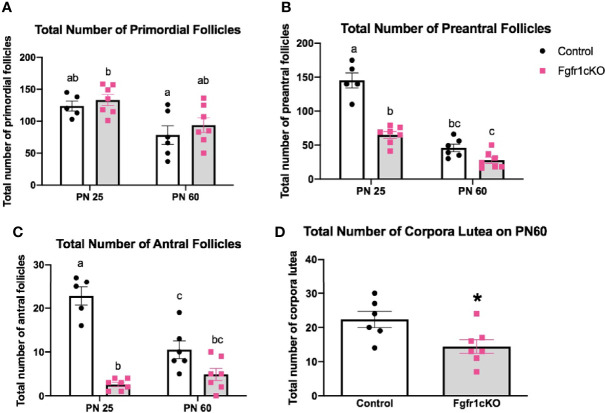
Ovarian morphology in PN25 and PN60 control and *Fgfr1cKO* female mice. Total number of primordial follicles **(A)**, preantral follicles **(B)**, and antral follicles **(C)** at PN 25 and PN60 (± 5) and total number of corpora lutea **(D)** at PN60 (± 5) were assessed by an investigator blind to the identity of the samples. All PN60 females were sacrificed on diestrus. Each bar represents mean ± SEM, *n* = 5–7. Two-way ANOVA results are indicated in Results section. Different letters above the bars indicate P < 0.05 by post-hoc test.

**Figure 7:**
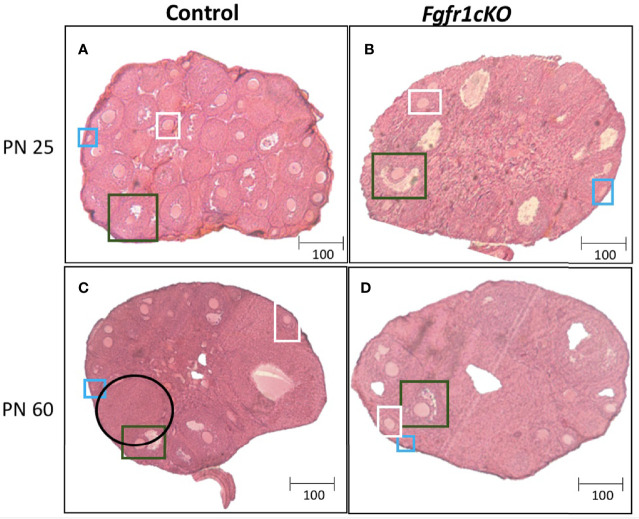
Representative photomicrographs of ovarian morphology in PN25 **(A, B)** and PN60 (± 5 days; **C, D**) control **(A, C)** and Fgfr1cKO **(B, D)** females. All PN60 females were sacrificed on diestrus. Representative primordial follicles (blue box), preantral follicles (white box), antral follicles (green box), and corpus luteum (black circle) were indicated. Scale bars = 100 µm.

For the assessment of female gonadal histology, prominent differences were observed across age and genotype groups. Total number of primordial follicles exhibited an age effect [*F*(1,21)=14.3, *P*=0.0011] only (**Figure 6A**). However, preantral follicles (**Figure 6B**) and antral follicles (**Figure 6C**) both exhibited a significant effect of genotype ([*F*(1,21)=58.17, *P*<0.0001] for preantral follicles; [*F*(1,21)=72.6, *P*<0.0001] for antral follicles), age ([*F*(1,21)=111.7, *P*<0.0001] for preantral follicles; [*F*(1,21)=10.87, *P*<0.0034] for antral follicles), and genotype x age interaction ([*F*(1,21)=23.3, *P*<0.0001] for preantral follicles; [*F*(1,21)=23.07, *P*<0.0001] for antral follicles). Post-hoc test showed significantly reduced preantral and antral follicles in the ovaries of PN25 *Fgfr1cKO* females compared to age-matched controls (**Figures 6B, C**), but these two follicle types were not affected in older PN60 *Fgfr1cKO* females compared to controls (**Figures 6B, C**). Lastly, we measured the number of corpora lutea in PN60 females only (**Figure 6D**) since PN25 females had not yet begun their estrous cycle. A significant reduction in the number of corpora lutea was observed in PN60 *Fgfr1cKO* compared to control females (**Figure 6D**). Photomicrographs (**Figure 7**) demonstrated the marked reduction in the number of antral and preantral follicles in a PN25 *Fgfr1cKO* ovary (**Figure 7B**) compared to control (**Figure 7A**) and the reduced presence of corpora lutea in PN60 *Fgfr1cKO* ovary (**Figure 7D**) compared to control (**Figure 7C**).

Because corpora lutea were disrupted in PN60 *Fgfr1cKO* females, we further assessed the reproductive performance of sexually mature control and *Fgfr1cKO* females between PN40-240. Time to first litter, total number of pups produced, days between litters, and pups per litter were not different between control and *Fgfr1cKO* females (**Supplementary Figure 4**). Male fertility was not examined since *Fgfr1cKO* males lacked a gonadal phenotype on PN60.

## Discussion

In this study, we have shown that a conditional deletion of *Fgfr1* in GnRH neurons disrupted several aspects of the HPG axis, with the most notable impacts occurring in the gonads of PN25 mice. However, these disruptive impacts became largely undetectable as the animals aged. Our results indicate Fgfr1 signaling directly on GnRH neurons has only minor effects during early puberty within a limited period and does not significantly alter adult reproduction.

The GnRH system, measured by *GnRH* transcript and peptide, was not significantly disrupted in *Fgfr1cKO* mice of both sexes and age groups (**Figures 1**–**3**). The lack of a GnRH system phenotype in *Fgfr1cKO* mice contrasts sharply with the severe GnRH insufficiency observed in humans and mice harboring global *Fgfr1* gene inactivation (11, 24). In fact, both human and mouse studies have shown that global *Fgfr1* deficiency leads to a failure of GnRH neurons to undergo migration or fate specification (11, 25). These significant GnRH system deficits were not observed in our current study due to two notable differences between the global and conditional deletion models. First, since the conditional deletion of *Fgfr1* could only occur in specified GnRH neurons with activated GnRH promoter, Fgfr1 signaling in GnRH progenitors could not be disrupted in our mice. In other words, if Fgfr1 signaling acted predominantly on progenitor cells to drive GnRH neuron fate specification, we would not be able to detect GnRH system defects in *Fgfr1cKO* mice. Second, *Fgfr1* is expressed abundantly in the epithelial and mesenchymal components of the olfactory pit, where nascent GnRH neurons emerged (12, 13), and in the GnRH neuron migratory pathway along the nasal septum and in the olfactory ensheathing cells (26). *Fgfr1* is also abundantly expressed in neuroglial cells with potential paracrine and physical access to GnRH neurons (27–30). Fgfr1 signaling in these surrounding regions is thought to critically support and guide the development and function of GnRH neurons, and such a role of Fgfr1 would not be disrupted in our conditional deletion model.

Despite the aforementioned possibilities for why *Fgfr1cKO* mice may lack a GnRH system phenotype, the seemingly normal GnRH system in these mice was somewhat unexpected for several reasons. First, potent neurotrophic effects of Fgf signaling were reported for immortalized GnRH neuronal cell lines and primary GnRH neurons in culture (13, 31, 32), and the attenuation of Fgfr1 function resulted in abnormal cellular morphology and survival in the former (33). Further, a mouse model (dnFGFR mouse) expressing a mutant dominant-negative Fgfr in GnRH neurons exhibited a significant disruption of the GnRH system, puberty, and reproductive function (33). It is at present unclear why *Fgfr1cKO* mice lack a detectable GnRH system defect and exhibit a much milder reproductive phenotype than dnFGFR mice. One possibility is that since GnRH neurons also express *Fgfr3* (13), a receptor with Fgf ligand selectivity overlapping Fgfr1 (34), *Fgfr3* could be upregulated to compensate for the lack of *Fgfr1* in *Fgfr1cKO* mice. This compensation would not be possible in dnFGFR mice since the mutant receptor disrupts the function of both Fgfr1 and Fgfr3 (33). Supporting this notion, the downregulation of *Fgfr1* consistently led to the upregulation of *Fgfr3* in a GnRH neuronal cell line, suggesting a reciprocal relationship between these two receptors to control the dosage of Fgf signaling (35). Notwithstanding this caveat, results from *Fgfr1cKO* mice suggest that Fgfr1 signaling directly on fate-specified GnRH neurons may not significantly impact GnRH mRNA and peptide accumulation, and is unlikely to be critical for the post-fate specification development and survival of the GnRH system.

Despite the seemingly normal GnRH system in *Fgfr1cKO* mice gauged by the *GnRH* transcript and peptide, the HPG axis of *Fgfr1cKO* mice was not entirely normal. A minor disruption in pituitary LH demonstrating a genotype x age interaction was observed in PN25 *Fgfr1cKO* males (**Figure 1C**), suggesting a greater vulnerability of younger males to pituitary LH disruption. More conspicuous gonadal disruptions were also observed in PN25 male and female *Fgfr1cKO* mice (**Figures 4**–**7**). Both PN25 *Fgfr1cKO* males and females exhibited stunted gonadal maturation (**Figures 4**–**7**); these gonadal disruptions could be considered as a transient delay in the early phase of puberty since they were no longer observed at PN60.

Loss-of-function mutations of *Fgfr1* in humans were previously reported to be associated with a host of reproductive deficits including constitutional delayed puberty (36–38). Heterozygous mice globally hypomorphic for *Fgfr1* also exhibited delayed puberty (39). Further, female mice deficient in *HS6ST1*, an enzyme needed for the production of heparan sulfate, a critical coreceptor for Fgfr1 signaling, exhibited delayed puberty despite a seemingly normal GnRH neuronal population (40). It is at present unclear why the effects of conditional *Fgfr1* deletion on gonads were transient and observed only at PN25. Mammalian puberty is a continuous process spanning a period of days, with distinct anatomical and gonadal benchmarks observed along the way (41). The appearance of pubertal benchmarks, such as antral follicles, in mice has been reported as early as PN14 (42) and coincided with a gradual transition of GnRH release from an immature pattern in one-week old mice to the mature adult pattern suitable for stimulating gonadotropins (43). One possibility is that GnRH release in *Fgfr1cKO* mice may exhibit disrupted transition during this vulnerable period leading to puberty, resulting in delayed gonadal maturation. Our results highlight the complexity of how the function of a GnRH system can be disrupted in the absence of discernible changes in GnRH peptide and mRNA levels. Indeed, the GnRH system has been shown to be plastic and can compensate for GnRH peptide level even when the system is developmentally compromised (20).

Both male and female *Fgfr1cKO* mice exhibited transiently disrupted gonadal maturation. A previous study reported significantly decreased gonocyte proliferation in PN5 *hpg* male mice, leading to reduced spermatogonia by PN20 (44). The report suggested that GnRH was required for the stimulation of gonocytes during the neonatal period to expand the initial pool of spermatogenic cells (44, 45). As such, secretory defects in the GnRH system of *Fgfr1cKO* males may lead to reduced spermatogonia and transiently delay the appearance of mature spermatozoa in PN25 testes. Female ovarian maturation is more complex and requires stage-dependent changes in the secretory patterns of GnRH and gonadotropins (46–48). Control and *Fgfr1cKO* females were born with similar numbers of primordial follicles, a parameter independent of GnRH or gonadotropin function (49). However, decreased preantral and antral follicles in PN25 *Fgfr1cKO* females may reflect a compromised ability of primordial follicles to (1) initiate early growth or (2) persist after becoming antral follicles (49). Although only the latter process is GnRH-dependent, the timeframe of ovarian disruption is consistent with our hypothesis that *Fgfr1cKO* mice may experience a disrupted transition of GnRH release pattern in the period leading to puberty (43). The recovery of gonadal function in PN60 *Fgfr1cKO* mice may reflect the plasticity of the GnRH system (50) or the ability or the pituitary or gonad to compensate for the GnRH system defect.

The only reproductive deficit in *Fgfr1cKO* mice that persisted into PN60 was the reduction of corpora lutea in females (**Figure 6D**), but this deficit did not alter female fertility between PN40-240 (**Supplementary Figure 4**). Corpora lutea in mice can persist for up to 4 cycles in the absence of pregnancy (51). As such, a reduction in corpora lutea in PN60 *Fgfr1cKO* females may reflect an accumulation of ovarian defects before PN60 instead of ongoing ovarian defects in PN60 or older females.

Overall, our results showed that a conditional deletion of *Fgfr1* in GnRH neurons impacted the HPG axis only during a limited period of time coinciding with early puberty. These impacts were primarily upon the gonads, transient, and mostly undetectable by PN60. As such, the severely defective GnRH neuronal system seen in humans and mice harboring global *Fgfr1* deficiency may be due to the disruption of GnRH progenitors or tissues supporting GnRH neurons rather than GnRH neurons themselves. A caveat was that we have only validated the parental lines and did not directly measure the extent of *Fgfr1* deletion in GnRH neurons. Such measurement has been made difficult by the extracellular portion of Fgfr1 protein that still remained after the recombination (16) and the short target sequence available for detecting the recombination of a very low abundance transcript. As such, we cannot rule out the possibility that the deletion of *Fgfr1* in GnRH neurons of *Fgfr1cKO* mice may have been incomplete. Future challenges on the HPG axis, including gonadectomy, gonadal steroid administration, stress, and aging, may also reveal additional reproductive deficits in *Fgfr1cKO* mice.

## Data Availability Statement

The raw data supporting the conclusions of this article will be made available by the authors, without undue reservation.

## Ethics Statement

The animal study was reviewed and approved by the Institutional Animal Care and Use Committee at the University of Colorado Boulder.

## Author Contributions

CD and P-ST conceived and designed the experiments. CD, CH, KS, and NA performed the experiments. CD and P-ST performed data and statistical analysis. CD and P-ST wrote the manuscript. All authors contributed to the article and approved the submitted version.

## Funding

This work was supported by NIH R01 HD083260 to P-ST.

## Conflict of Interest

The authors declare that the research was conducted in the absence of any commercial or financial relationships that could be construed as a potential conflict of interest.
