# Supplementary material for: Conditional Fgfr1 Deletion in GnRH Neurons Leads to Minor Disruptions in the Reproductive Axis of Male and Female Mice
Source: Front Endocrinol (Lausanne). 2021 Feb 19;11:588459. doi: 10.3389/fendo.2020.588459 (PMC7933197; doi:10.3389/fendo.2020.588459)
Supplement: Supplementary file 1 [file Table_1.docx]

## Supplementary Table 1. Assessment of pubertal onset in male and female control and *Fgfr1cKO* mice.

|  | Control | *Fgfr1cKO* | P value (Student's *t*-test) |
| --- | --- | --- | --- |
| Age of BPS (days) | 30.0 ± 0.79 (n = 10) | 29.3 ± 0.92 (n = 10) | 0.42 |
| Age of VO (days) | 37.92 ± 1.41 (n = 12) | 38.2 ± 1.46 (n = 10) | 0.89 |
| Age of first estrus (days) | 42.6 ± 1.22 (n = 10) | 43.1 ± 1.42 (n = 10) | 0.79 |


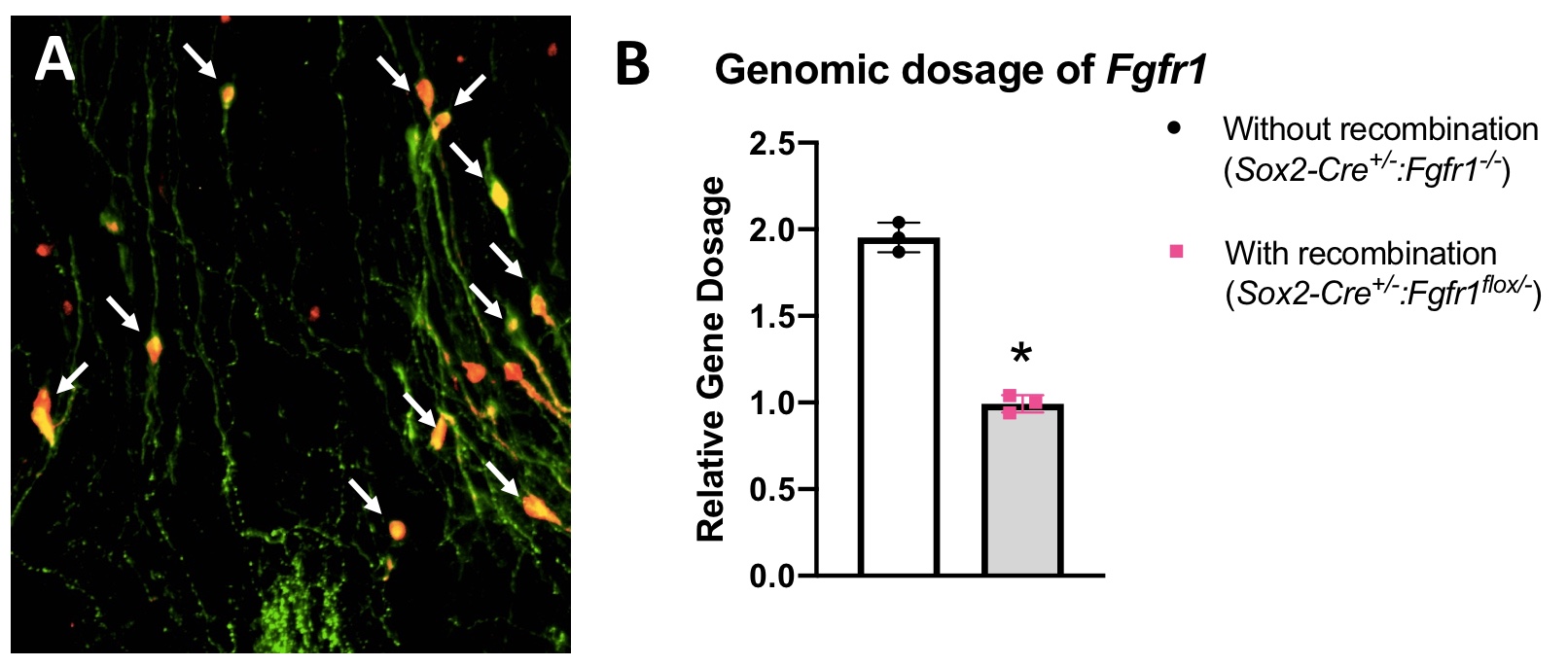


Supplementary Figure 1. *GnRH-Cre^+/-^* **(A)** and *Fgfr1^flox/flox^* **(B)** mice used in the present study were validated separately. **(A)** GnRH-immunopositive neurons (green) in a mouse with the expression of tdTomato (red) targeted to GnRH neurons using the *GnRH-Cre^+/-^* mouse. The expression of tdTomato was driven by the Cre mediated excision of a stop codon upstream of the tdTomato transgene, thus the overlap between tdTomato and GnRH (yellow, indicated with white arrows) demonstrated the effective targeting of *Cre* expression in *GnRH-Cre^+/-^* mice. **(B)** The dosage of *Fgfr1* Exon 4 in the genomic DNA was halved when a deleter mouse (*Sox2-Cre^+/-^*) was used to excise *Fgfr1* Exon 4 in *Fgfr1^flox/-^ mice,* demonstrating the effectiveness of the *Fgfr1* floxed allele in Cre-mediated recombination.


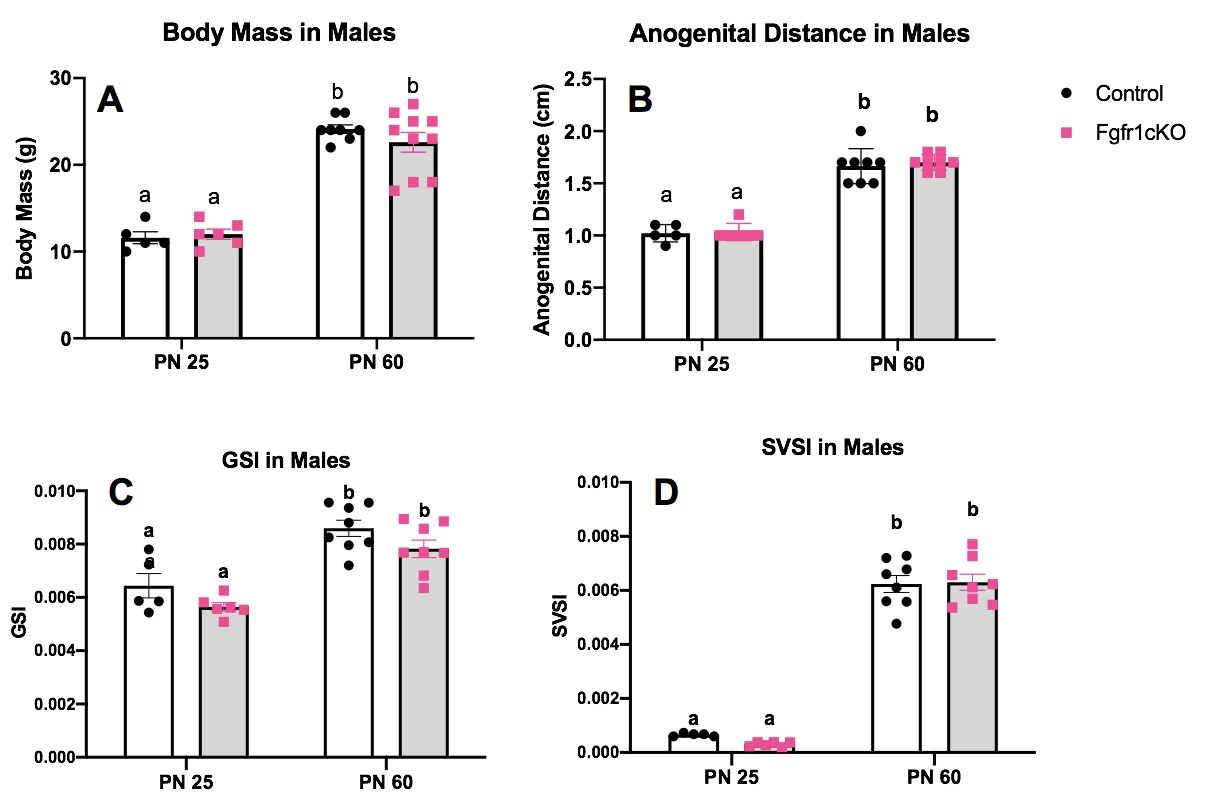


Supplementary Figure 2. Body mass **(A)**, anogenital distance **(B)**, GSI (gonadosomatic index) **(C)**, and SVSI (seminal vesicle somatic index) **(D)** in male PN25 and PN60 control and *Fgfr1cKO* mice. Each bar represents mean ± SEM, *n* = 5-10. Different letters above the bars indicate P < 0.05 by post-hoc test.


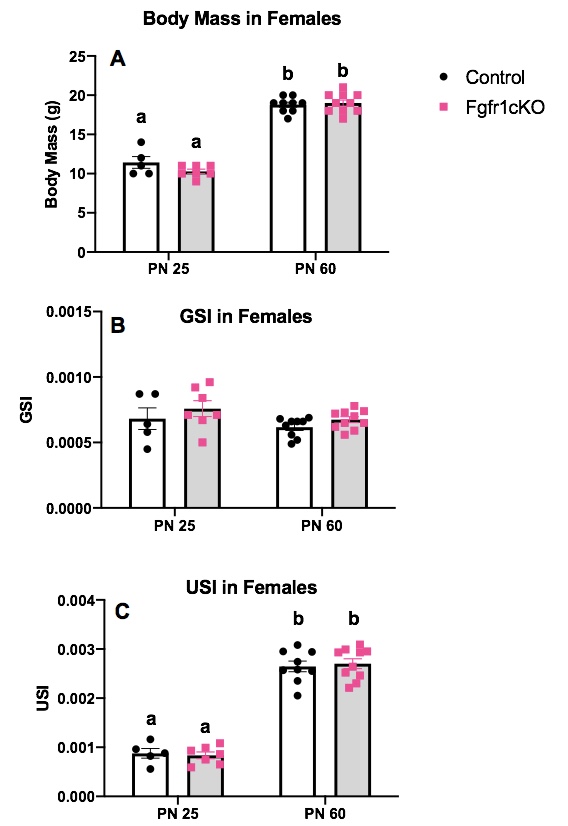


Supplementary Figure 3. Body mass **(A)**, GSI (gonadosomatic index) **(B)**, and USI (uterine somatic index) **(D)** in PN25 and PN60 female control and *Fgfr1cKO* mice. Each bar represents mean ± SEM, *n* = 5-10. Different letters above the bars indicate P < 0.05 by post-hoc test.


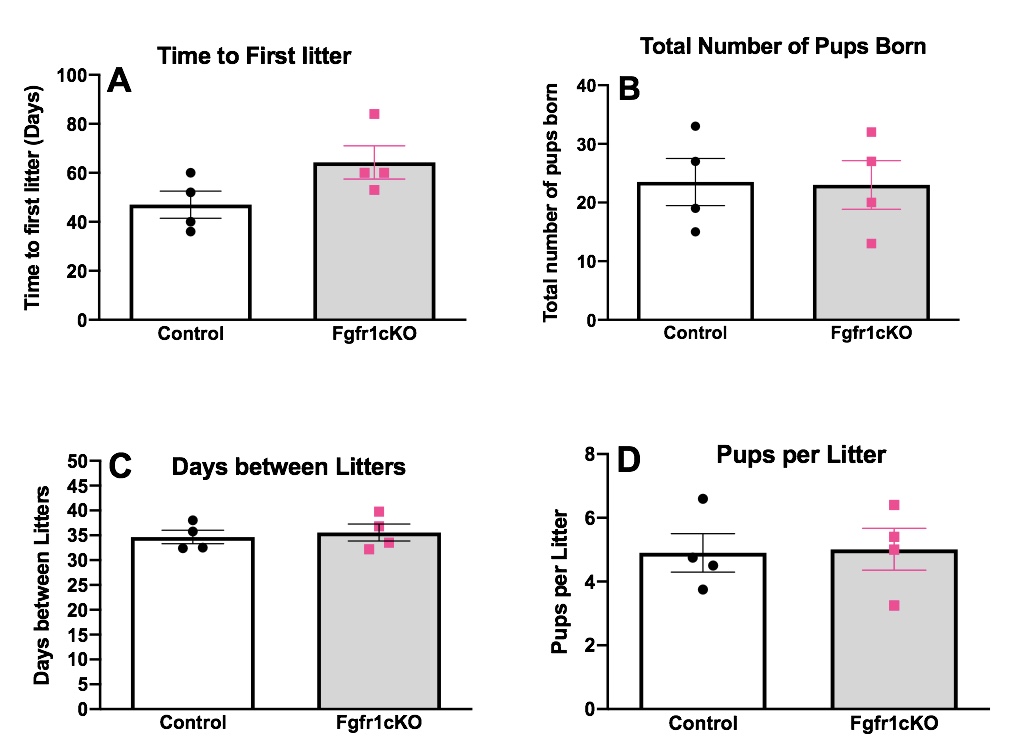


Supplementary Figure 4. Fertility assessment of female control and *Fgfr1cKO* mice between PN40-PN240. No significant differences were observed between genotypes in latency to first litter **(A)**, total number of pups born **(B),** average days between litters **(C)**, and average number of pups per litter **(D)**. Each bar represents mean ± SEM, *n* = 4.
